# Clinical significance of serum levels of 14-3-3β protein in patients with stable chronic obstructive pulmonary disease

**DOI:** 10.1038/s41598-023-32096-4

**Published:** 2023-03-24

**Authors:** Decai Wang, Lizong Rao, Huiren Lei, Wencui Li, Qiufang Yu, Wei Li, Jianghong Wei, Shuyun Xu, Biwen Mo

**Affiliations:** 1 Department of Respiratory and Critical Care Medicine, Key Laboratory of Pulmonary Diseases of Health Ministry, Key Site of National Clinical Research Center for Respiratory Disease, Tongji Hospital, Tongji Medical College, Huazhong University of Science and Technology, Wuhan, 430030 Hubei China; 2 Department of Respiratory and Critical Care Medicine, Guangxi Zhuang Autonomous Region Education Department Key Laboratory of Respiratory Diseases, Guangxi Health Commission Key Laboratory of Glucose and Lipid Metabolism Disorders, Second Affiliated Hospital of Guilin Medical University, Guilin, 541004 Guangxi China; 3 Department of Respiratory and Critical Care Medicine, Affiliated Hospital of Guilin Medical University, Guilin, 541004 Guangxi China; 4 Key Laboratory of Respiratory Disease of Zhejiang Province, Department of Respiratory and Critical Care Medicine, Second Affiliated Hospital of Zhejiang University School of Medicine, Hangzhou, 310000 Zhejiang China

**Keywords:** Respiration, Inflammation

## Abstract

Nowadays, the diagnosis and treatment of COPD are often based on the results of lung function tests. Certain individuals, however, are not candidates for lung function testing due to pulmonary bullae, cardiac failure, low lung function, and other factors. Therefore, we evaluated whether serum tyrosine3-monooxygenase/tryptophan5-monooxygenase activation protein β (14-3-3β) could be a biomarker for the diagnosis of stable COPD patients. The expression of serum 14-3-3β protein was evaluated by an enzyme-linked immunosorbent assay. The association between its concentrations and clinical parameters of stable COPD patients were analyzed by correlation analysis and ROC curve. The results before propensity score matching (PSM) showed that serum 14-3-3β protein concentrations (ng/ml) in stable COPD patients were significantly higher than in healthy controls (*P* < 0.001). Furthermore, serum 14-3-3β protein concentrations were higher in GOLD 3&4 COPD patients compared with healthy participants, GOLD 1 and GOLD 2 COPD patients (*P* < 0.05), which shows that the concentration of 14-3-3β protein correlates with disease severity in stable COPD patients. After 1:1 PSM, there was also a statistically significant rise in 14–3-3 protein levels in stable COPD patients compared to healthy controls (*P* < 0.01). Serum 14-3-3β protein levels were positively correlated with blood neutrophil levels (*P* < 0.05), and negatively related to lung function parameters in stable COPD patients (*P* < 0.01). When the cutoff value was set at 29.53 ng/ml, the ROC curve yielded a sensitivity of 84.9% and a specificity of 68.3% for diagnosing stable COPD. The 14-3-3β protein may be a potential serum biomarker for the diagnosis of stable COPD patients, which is associated with disease severity, systemic inflammation, and small airway obstruction.

## Introduction

Chronic obstructive pulmonary disease (COPD) is defined as a clinical syndrome characterized by chronic respiratory symptoms, structural pulmonary abnormalities, lung function impairment, or any combination of these^1,2^. Long-term exposure to other lung irritants–such as air pollution; chemical fumes; dust; and indoor products of biomass fuels, such as wood burned in stoves–may contribute to COPD^3^. A rare genetic condition called α_1_-antitrypsin deficiency can also cause the disease^1^. COPD affects close to 400 million people and is already the third leading cause of death worldwide^1^. The China Pulmonary Health Study (CPHS) shows that the prevalence of COPD among adults aged 20 and over is 8.6% and as high as 13.7% over 40 years old, which estimated that the number of COPD patients is close to one billion in China^4^. COPD causes substantial health and economic burdens. Currently, the diagnosis and treatment of COPD are often based on lung function test results^1^. However, some patients are not suitable for lung function tests because of pulmonary bullae, cardiac insufficiency, low lung function, suboptimal effort, active bleeding, and so forth. Hence, identifying new biomarkers that can enhance the detection rate in patients with COPD has been a hot research topic.

The 14–3-3 protein family is constituted by 28–33 kDa acidic proteins^5^ found in all eukaryotes that play a critical role in the regulation of intracellular functions including protein synthesis, cellular metabolism, protein trafficking, signal transduction, and cytoskeletal transport^6–8^. In mammalian cells, 14–3-3 protein has seven isoforms (α/β, ε, η, γ, σ, θ/τ, and δ/ζ), with α and δ representing the phosphorylated versions of β and ζ, respectively^9^. The 14–3-3 protein family is a class of protein able to interact with a multitude of targets by establishing protein–protein interactions (PPIs)^7^. Previous evidence shows that misregulation of 14–3-3 protein contributes to important human diseases such as cancer, neurodegenerative disorders, and infection by Giardia intestinalis^10–14^. However, current studies have found that the 14–3-3 protein family subtypes interact with target proteins to participate in the pathogenesis of varieties of autoimmune diseases, including rheumatoid arthritis, systemic sclerosis, and large vessel vasculitis^15–17^. Furthermore, our previous research found that an increase in the level of serum 14-3-3β protein may be associated with airway inflammation and lung function decline in the acute exacerbation of asthma^18^. Therefore, the 14–3-3 protein family plays an important role in the development and progression of immune inflammation.

Pulmonary inflammation, oxidative stress, protease and anti-protease imbalance play important roles in the pathogenesis of COPD^19^. Inflammatory cells, epithelial cells, and other structural cells release a variety of inflammatory mediators, attract circulating inflammatory cells, amplify the inflammatory process, and induce changes in lung tissue structure^20^. Studies suggest that chronic airway inflammation in COPD involves multiple cytokines, which cause high secretion of mucus and obstruction of small airways, as well as damage to the lung parenchyma, and thus resulting in restricted airflow and excessive lung inflation^21^. Hence, the aim of this study was to analyze the changes in the serum concentrations of 14-3-3β protein in patients with stable COPD and explore the associations between their concentrations and clinical parameters of stable COPD.

## Results

### The clinical characteristics and clinical parameters were matched via PSM analysis

Before PSM, seventy-three patients with stable COPD and 63 healthy controls were enrolled. The average age was 66.4 years (range, 21–80 years) for stable COPD patients and 40 years (range, 30–70 years) for healthy controls. Sixty-two (85%) stable COPD patients and 25 (40%) healthy controls were male. After 1:1 PSM, 9 patients from each group (stable COPD and healthy controls) were enrolled in the analysis. There were no significant differences in clinical characteristics (age, gender, BMI) between the two groups. Clinical parameters (blood neutrophil ratio, blood neutrophil count, lung function parameters) with before or after matching for all participants were given in **Table ****1**.

**Table 1:** Demographics and clinical baseline characteristics.

Characteristic	Original cohort (n = 136)		*P*	Matched cohort (n = 18)		*P*
	HCs group (n = 63)	Stable COPD group (n = 73)		HCs group (n = 9)	Stable COPD group (n = 9)	
Sex (M), n(%)	25 (40)	62 (85)	< 0.001	5 (56)	5 (56)	0.681
Age (years)	39.95 ± 13.64	66.38 ± 10.15	< 0.001	55.67 ± 7.43	55.78 ± 5.47	0.972
BMI (kg/m^2^)	22.78 ± 3.07	22.10 ± 3.80	0.261	24.11 ± 4.29	23.37 ± 3.98	0.707
Neutrophil ratio	0.58 ± 0.08	0.64 ± 0.11	0.002	0.57 ± 0.10	0.61 ± 0.10	0.473
Neutrophil count (4 × 10^9^/L)	3.83(2.91–4.58)	4.34(3.48–7.09)	0.002	4.14(2.52–4.62)	3.70(3.39–5.16)	0.730
14-3-3β (ng/mL)	25.77(18.09–34.41)	40.81(30.98–53.82)	< 0.001	25.77(22.48–29.04)	36.25(30.12–41.33)	0.003
FVC (L)	3.27 ± 0.82	2.31 ± 0.86	< 0.001	3.31 ± 1.10	2.49 ± 0.88	0.102
FVC % pred (%)	92.96 ± 12.33	71.22 ± 19.82	< 0.001	98.26 ± 14.55	76.98 ± 19.65	0.019
FEV1 (L)	2.82 ± 0.73	1.32 ± 0.66	< 0.001	2.74 ± 0.93	1.47 ± 0.69	0.005
FEV1% pred (%)	94.45 ± 12.91	51.65 ± 22.83	< 0.001	99.67 ± 13.48	55.21 ± 22.30	< 0.001
FEV1/FVC (%)	86.09 ± 5.39	55.78 ± 12.15	< 0.001	82.95 ± 6.03	57.79 ± 12.62	< 0.001
FEF25 (L)	1.88(1.10–3.27)	0.24(0.17–0.40)	< 0.001	1.88(0.85–4.62)	0.31(0.16–0.45)	< 0.001
FEF25%pred (%)	85.42 ± 24.02	25.15 ± 19.37	< 0.001	87.18 ± 29.88	19.67 ± 9.88	< 0.001
FEF50 (L)	3.75(2.97–4.98)	0.59(0.40–1.30)	< 0.001	3.24(2.51–5.45)	0.98(0.42–1.86)	< 0.001
FEF50% pred (%)	93.86 ± 25.68	25.14 ± 18.58	< 0.001	102.77 ± 36.35	29.57 ± 21.18	< 0.001
MMEF (L)	3.30 ± 1.10	0.75 ± 0.70	< 0.001	3.13 ± 1.39	0.78 ± 0.50	< 0.001
MMEF% pred (%)	93.23 ± 28.53	24.48 ± 17.73	< 0.001	102.54 ± 37.04	25.07 ± 14.54	< 0.001

Results are represented as n, mean with SD or median (IQR). Data were tested by χ2 testing, ANOVA, or Kruskal–Wallis test. *HCs* Healthy controls; *BMI* Body mass index; *FVC* Forced vital capacity; *FEV1* Forced expiratory volume in one second; *FEV1/FVC* The ratio of forced expiratory volume in one second to forced vital capacity; *FEF25* Forced expiratory flow 25% of FVC; *FEF50* Forced expiratory flow 50% of FVC; *MMEF* maximal mid-expiratory flow; % pred: % predicted.

A *P* value of less than 0.05 was statistically significant.

### Serum 14-3-3β protein expression levels in healthy participants and stable COPD patients

**Figure 1:**
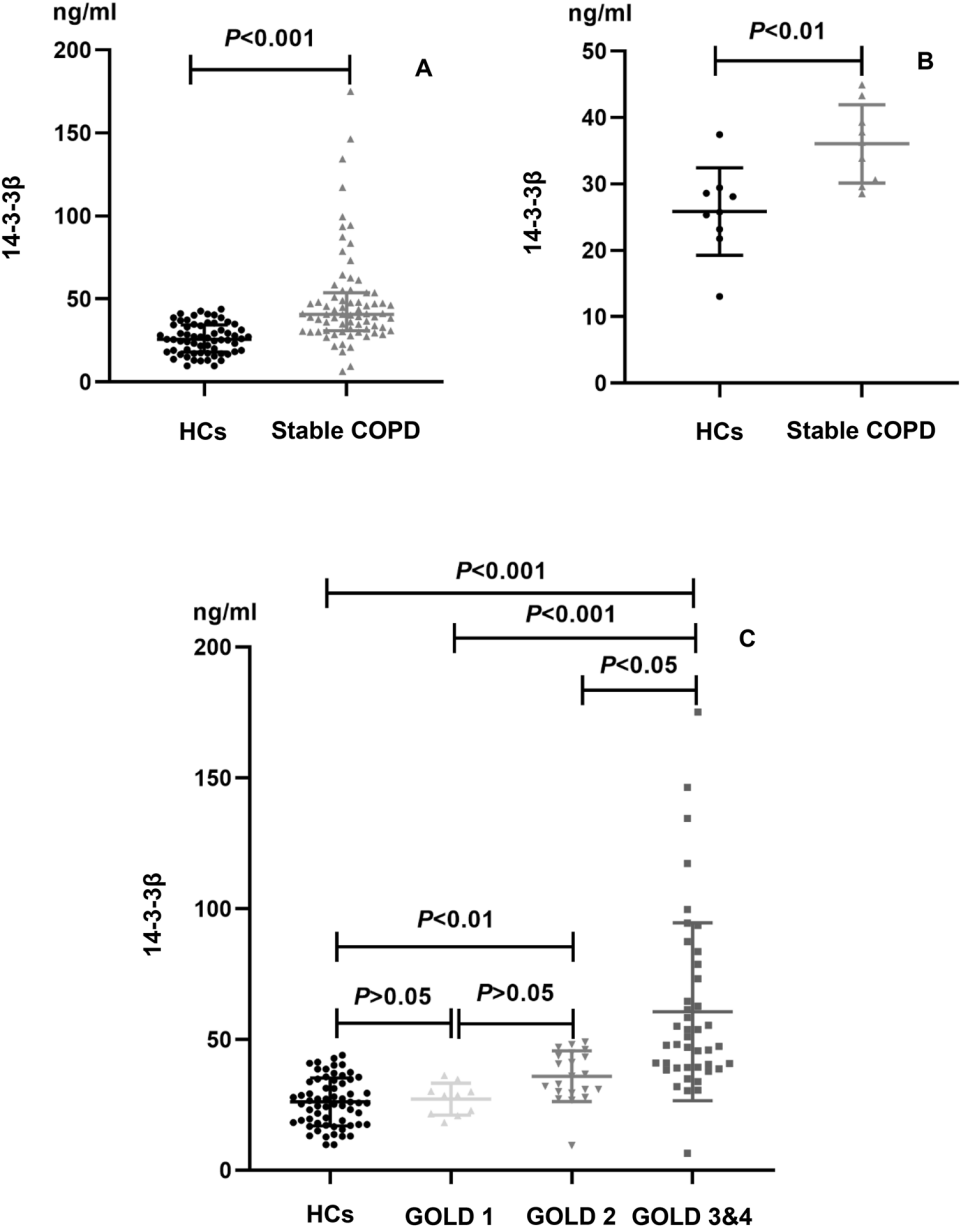
(**A**, **B**) Comparison of serum 14-3-3β protein levels in healthy controls (HCs) and stable COPD patients before or after propensity score matching; (**C**) Comparison of serum 14-3-3β protein levels in the GOLD grade of COPD.

Before PSM, there was a statistically highly significant increase in the median (interquartile range, IQR) 14-3-3β protein concentrations (ng/ml) in stable COPD patients [40.81 ng/ml (30.98–53.82)] than in healthy controls [25.77 ng/ml (18.09–34.41); *P* < 0.001], as shown in Table 1 and Fig. 1A. After 1:1 PSM, our results also showed that the median (IQR) of serum 14-3-3β protein levels in group with stable COPD [36.25 ng/ml (30.12–41.33)] was much higher than that in healthy controls [25.77 ng/ml (22.48–29.04); *P* = 0.003], as shown in Table 1 and Fig. 1B. In addition, serum levels of 14-3-3β protein were significantly elevated in patients with GOLD 3&4 COPD compared with healthy participants, GOLD 1 and GOLD 2 COPD patients [47.98 ng/ml (39.27–74.57) versus 25.77 ng/ml (18.09–34.41), 28.62 ng/ml (21.48–31.34), and 36.05 ng/ml (29.84–44.38), *P* < 0.05, respectively, Fig. 1C and Supplementary Table 1]. Finally, we compared serum 14-3-3β protein levels between GOLD 1 and GOLD 2 COPD patients, and the results showed no statistically significant difference between the two groups (*P* > 0.05, Fig. 1C and Supplementary Table 1).

### Correlation of serum 14-3-3β protein with clinical parameters

**Figure 2:**
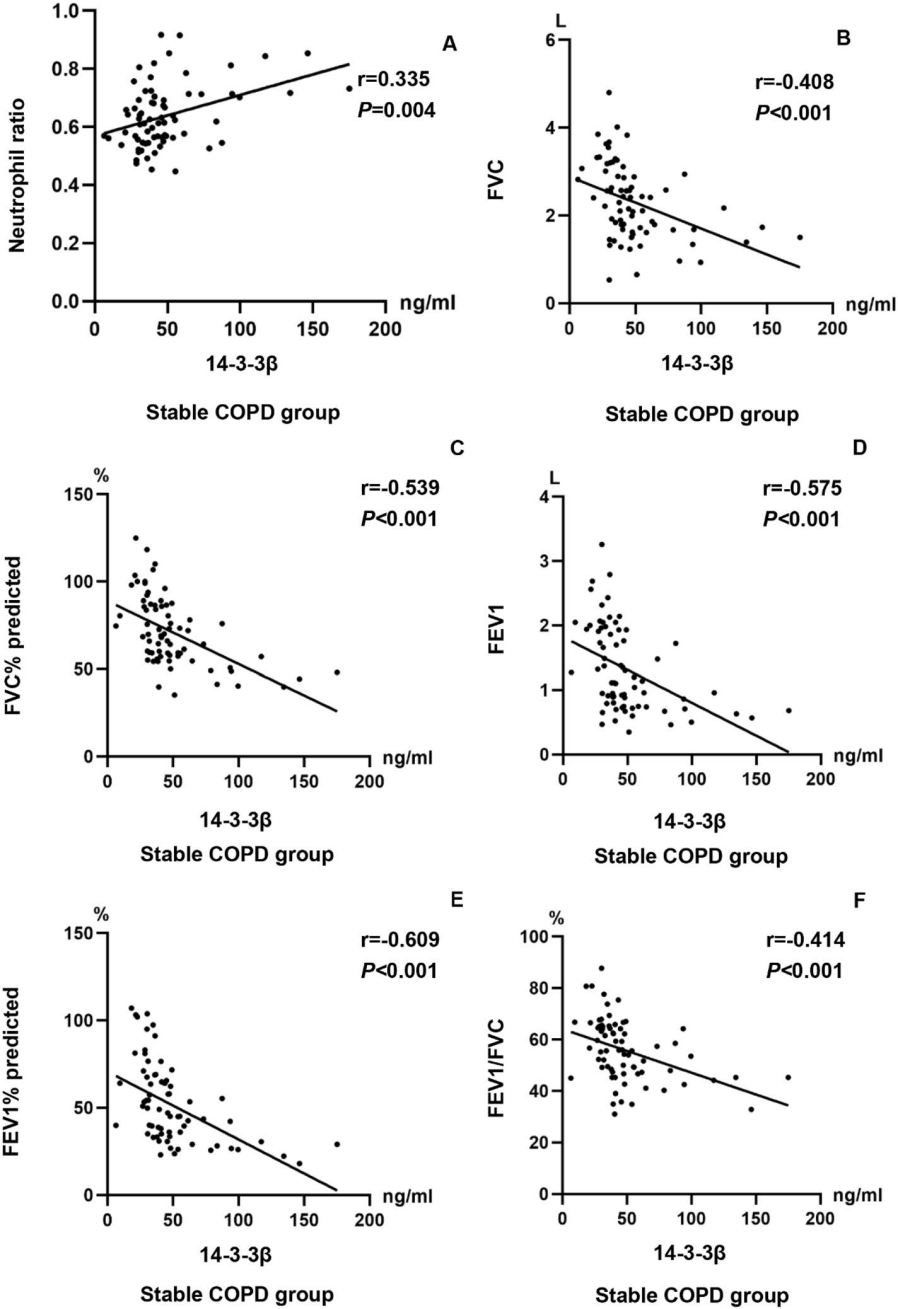

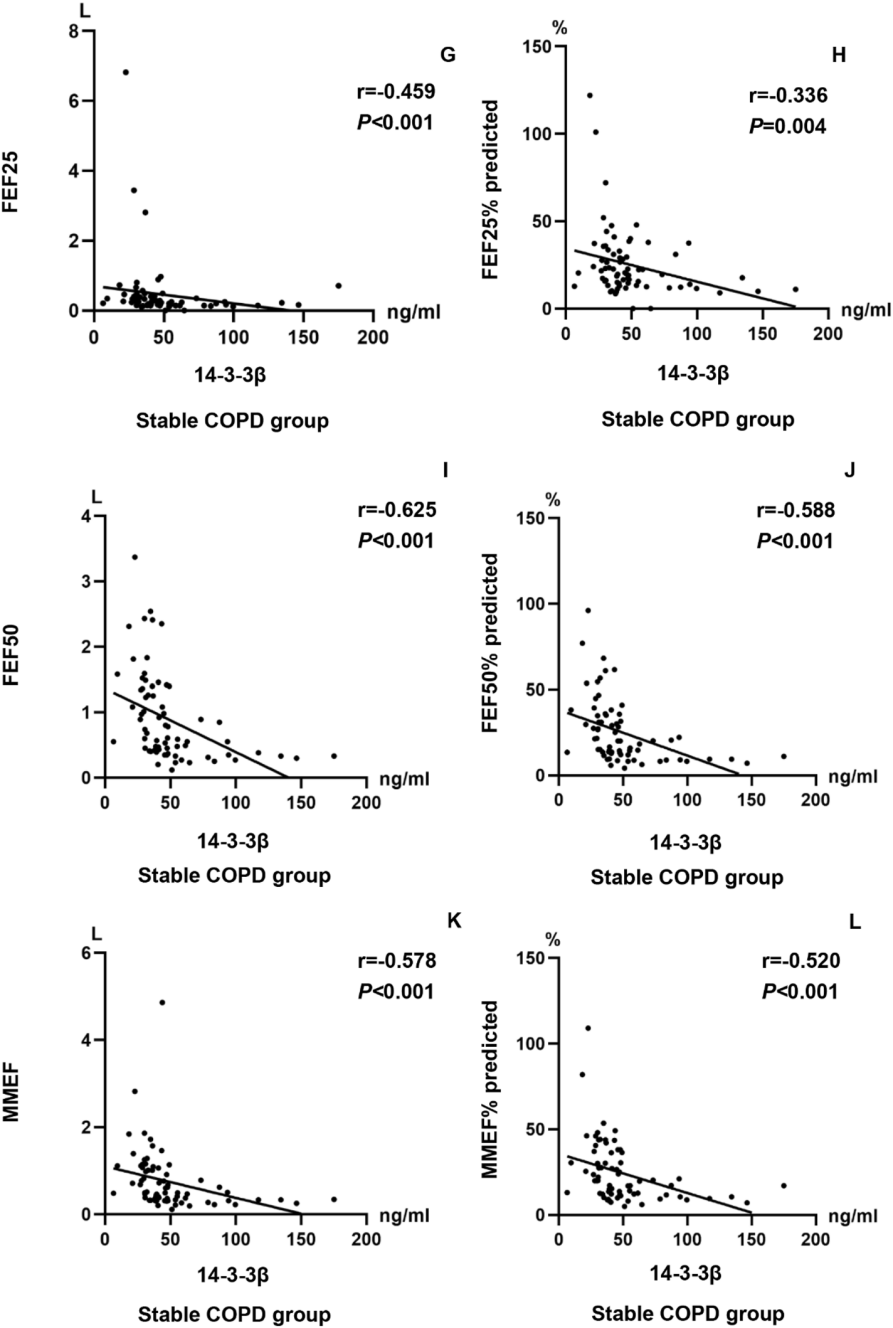
Correlation between serum 14-3-3β protein levels and clinical parameters in stable COPD patients. (**A**–**L**)**.** Correlation between 14-3-3β protein and blood neutrophil ratio, FVC, FVC% predicted, FEV1, FEV1% predicted, FEV1/FVC, FEF25, FEF25% predicted, FEF50, FEF50% predicted, MMEF, and MMEF % predicted in stable COPD patients.

The correlation matrices presented in Table 2 illustrate the relationship between the levels of serum 14-3-3β protein and clinical parameters in stable COPD patients, respectively. As expected, the levels of serum 14-3-3β protein were positively correlated with peripheral blood neutrophil ratio (r = 0.335 *P* = 0.004) and peripheral blood neutrophil count (r = 0.266 *P* = 0.023), and negatively related to FVC(r = − 0.408 *P* < 0.001), FVC% predicted(r = − 0.539 *P* < 0.001), FEV1(r = − 0.575 *P* < 0.001), FEV1% predicted(r = − 0.609 *P* < 0.001), FEV1/FVC (r = − 0.414 *P* < 0.001), FEF25(r = − 0.459 *P* < 0.001), FEF25% predicted(r = − 0.336 *P* = 0.004), FEF50(r = − 0.625 *P* < 0.001), FEF50% predicted(r = − 0.588 *P* < 0.001), MMEF(r = − 0.578 *P* < 0.001), and MMEF% predicted(r = − 0.520 *P* < 0.001) in stable COPD patients, as shown in Fig. 2A–L.

**Table 2:** Association of 14-3-3β with clinical parameters in stable COPD patients.

Clinical parameters	14–3− 3β	
	*r*	*P*
Neutrophil ratio	0.335	0.004
Neutrophil count (4 × 10^9^/L)	0.266	0.023
FVC (L)	− 0.408	< 0.001
FVC % Pred (%)	− 0.539	< 0.001
FEV1 (L)	− 0.575	< 0.001
FEV1% pred (%)	− 0.609	< 0.001
FEV1/FVC (%)	− 0.414	< 0.001
FEF25 (L)	− 0.459	< 0.001
FEF25% pred (%)	− 0.336	0.004
FEF50 (L)	− 0.625	< 0.001
FEF50% pred (%)	− 0.588	< 0.001
MMEF (L)	− 0.578	< 0.001
MMEF% pred (%)	− 0.520	< 0.001

*FVC* forced vital capacity; *FEV1* forced expiratory volume in one second; FEV1/FVC: the ratio of forced expiratory volume in one second to forced vital capacity; *FEF25* Forced expiratory flow 25% of FVC; *FEF50* Forced expiratory flow 50% of FVC; *MMEF* maximal mid-expiratory flow; % pred: % predicted. A P value of less than 0.05 was statistically significant.

### Receiver operating characteristic (ROC) curves of 14-3-3β protein

**Figure 3:**
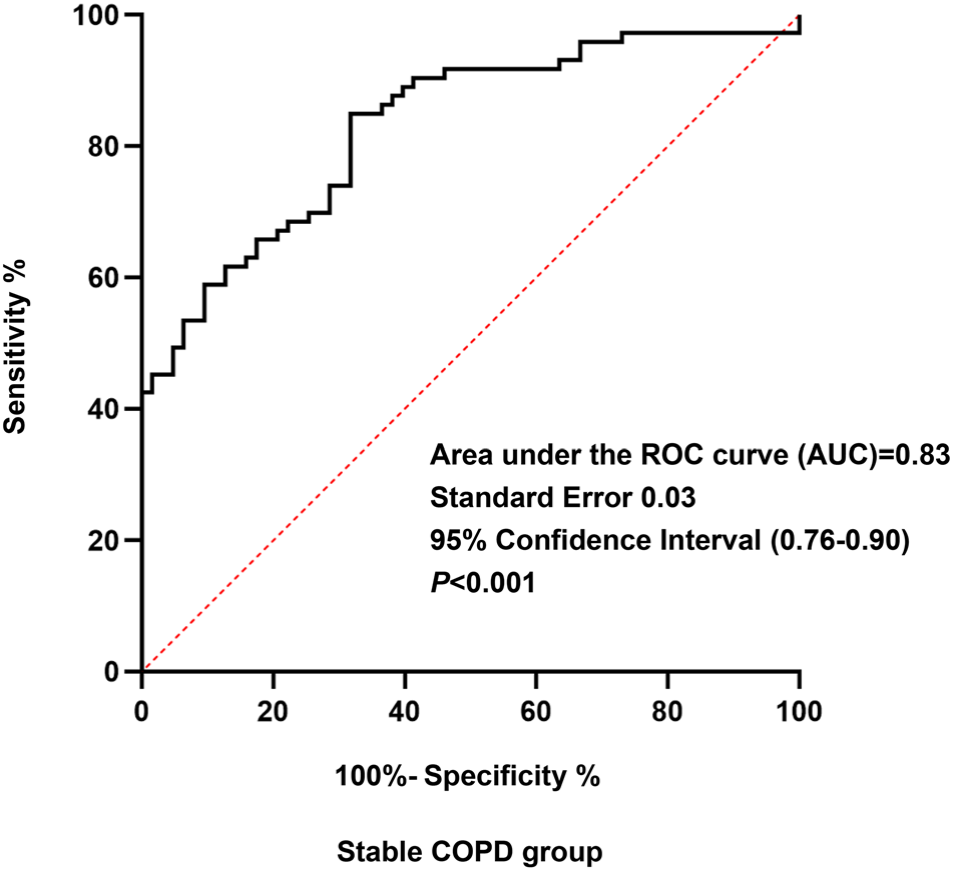
Receiver operating characteristic (ROC) curve for serum 14-3-3β protein levels comparing stable COPD patients with healthy controls.

ROC curve analysis comparing stable COPD patients with healthy subjects demonstrated a significant (*P* < 0.001) AUC of 0.83 (95% CI, 0.76–0.90), as shown in Fig. 3. When the cutoff value was set at 29.53 ng/ml, the ROC curve yielded a sensitivity of 84.9% and a specificity of 68.3% for diagnosing stable COPD.

### Multivariate analysis of serum 14-3-3β protein expression level

Through multivariate analysis, statistical results indicate FVC% pred (β = − 1.49, *P* = 0.006), FEV1(β = 2.11, *P* = 0.037), FEV1% predicted (β = 1.84, *P* = 0.018), FEV1/FVC (β = − 1.76, *P* < 0.001) and FEF50 (β = − 1.77, *P* = 0.021) were independent parameters associated with 14-3-3β protein, as shown in Table 3.

**Table 3:** Multivariate regression model of risk factors for serum 14-3-3β expression levels.

Parameter	B	SE	β	95% CI	*P*
Peripheral blood neutrophil ratio	16.31	30.39	0.07	− 43.90 ~ 76.51	0.593
Peripheral blood neutrophil count	0.41	1.35	0.04	− 2.26 ~ 3.07	0.761
Gender	− 14.59	7.96	− 0.27	− 30.36 ~ 1.19	0.070
Age	− 0.51	0.26	− 0.34	− 1.03 ~ 0.01	0.054
FVC	− 33.11	17.95	− 1.26	− 68.66 ~ 2.45	0.068
FVC% pred	− 1.87	0.67	− 1.49	− 3.20 ~ − 0.54	**0.006**
FEV1	53.00	25.05	2.11	3.39 ~ 102.62	**0.037**
FEV1% pred	1.63	0.68	1.84	0.29 ~ 2.97	**0.018**
FEV1/FVC	− 2.53	0.63	− 1.76	− 3.77 ~ − 1.28	** < 0.001**
FEF25	− 2.42	1.54	− 0.20	− 5.46 ~ 0.63	0.118
FEF25% pred	− 0.31	0.17	− 0.45	− 0.64 ~ 0.02	0.061
FEF50	− 24.68	10.55	− 1.77	− 45.59 ~ − 3.77	**0.021**
FEF50% pred	0.64	0.36	1.03	− 0.06 ~ 1.35	0.074
MMEF	3.12	5.24	0.19	− 7.26 ~ 13.49	0.553
MMEF% pred	0.30	0.22	0.49	− 0.14 ~ 0.74	0.183

Bolding indicates statistical significance. The value of B represents the unstandardized regression coefficient; The value of β represents the standardized regression coefficient. *SE* standard error; 95% CI: 95% confidence interval for B; *FVC* Forced vital capacity; *FEV1* Forced expiratory volume in one second; *FEV1/FVC* The ratio of forced expiratory volume in one second to forced vital capacity; FEF25: Forced expiratory flow 25% of FVC; *FEF50* Forced expiratory flow 50% of FVC; *MMEF* Maximal mid− expiratory flow; % pred: % predicted.

## Discussion

Chronic inflammation of COPD is a complex process involving various cytokines, inflammatory mediators, and immune cells^20^. Previous research has discovered that 14–3-3 protein can regulate T cell activation ^22^, Th2 cytokine production^16^ and Immunoglobulin E (IgE) class switching, and B cells antibody secretion^23^ via interactions with target proteins. Recent a study indicates that 14-3-3β protein may be associated with airway inflammation in the acute exacerbation of asthma^18^, but its roles in COPD are not known. The current observational study was designed to investigate the serum 14-3-3β protein level in stable COPD patients and to evaluate the relationship between the 14-3-3β protein level and systemic inflammation and small airway obstruction in stable COPD patients.

Our results suggest that patients with stable COPD have a significant increase in serum 14-3-3β protein levels compared with healthy subjects, which indicates that 14-3-3β protein may be involve in the development of stable COPD. Similarly, we compared serum 14–3-3 protein in healthy individuals to those in COPD patients with different GOLD grades. Serum levels of 14–3-3 proteins were considerably higher in patients with GOLD 2 and GOLD 3&4 COPD compared to healthy individuals, as illustrated in Fig. 1C and Supplementary Table1. What’s more, serum levels of 14-3-3β protein were significantly elevated in patients with GOLD 3&4 COPD compared with healthy participants, GOLD 1 and GOLD 2 COPD patients. In summary, it shows that the concentration of 14-3-3β protein correlates with disease severity in stable COPD patients.

Neutrophils have been linked to emphysematous alterations in COPD ^24^, such as through the release of neutrophil elastase^25^. Both innate and adaptive immunity are involved in the inflammatory response in COPD, with neutrophilic inflammation being the most prevalent inflammatory phenotype ^26,27^. In the present study, there was significant difference in peripheral blood neutrophil levels between stable COPD patients and healthy controls, with higher values in the former before PSM, which was consistent with previous studies by Gunay and colleagues^28^, However, there was no difference in blood neutrophil levels between stable COPD patients and healthy controls after PSM, which might be attributed to the small sample size after PSM. A recent study demonstrated that 14–3-3ζ protein may induce neutrophil migration to targets located at sites of infection or inflammation via p38 MAPK^29^. The levels of serum 14-3-3β protein were positively linked to peripheral blood neutrophil in stable COPD, suggesting that 14-3-3β protein may promote neutrophil migration to peripheral blood, thereby inducing a systemic inflammatory response. In this study, stable COPD patients showed significant differences in lung function parameters compared to healthy participants, which was in part agreement with previous studies by Hao and colleagues^30^. Also, the levels of serum 14-3-3β protein were inversely associated with lung function parameters in stable COPD patients. These findings demonstrate that 14-3-3β protein was associated with small airway obstruction and indicate that it may be an important risk factor for lung function decline in stable COPD.

ROC curve analysis comparing stable COPD patients with healthy controls demonstrated a significant difference in AUC. When the cutoff value was set at 29.53 ng/ml, the ROC curve yielded a sensitivity of 84.9% and a specificity of 68.3% for diagnosing stable COPD patients, suggesting that 14-3-3β protein might be a novel marker for the diagnosis of stable COPD patients.

Our study also has some potential limitations. Firstly, the sample size was relatively small to perform the necessary multi-factor analysis. Secondly, the included parameters for this study were relatively few. There was no in-depth study of whether the levels of 14-3-3β protein were different in sputum, bronchoalveolar lavage, airway epithelial cells, and lung tissue. In addition, we are unable to frequently measure 14-3-3β protein levels during the study period, which inhibits our ability to determine the effects of clinical intervention on 14-3-3β protein expression changes. Therefore, the further research is needed to clarify the practical application value of 14-3-3β protein as a new biomarker in a clinical setting.

## Conclusion

In summary, we demonstrated that stable COPD patients had higher serum 14-3-3β protein concentrations than healthy subjects and an increase in serum 14-3-3β protein concentrations may be associated with disease severity, systemic inflammation, and small airway obstruction in stable COPD patients, showing that 14-3-3β protein may play crucial roles in the pathogenesis of stable COPD. The AUC value of 14-3-3β protein was 0.83, which implied that it may be a potential serum biomarker.

## Methods

### Ethical statement

This study was a retrospective observational study and did not involve any form of therapeutic intervention. Written informed consent was obtained from all participants. The study was approved by the Ethics Committee of the Affiliated Hospital of Guilin Medical University (YJSLL202103). We confirm that all methods were performed in accordance with the relevant guidelines and regulations.

### Patients with stable COPD and healthy controls

This was an observational study that recruited 73 stable COPD patients from May 2020 to June 2021 from the outpatient department of the Department of Respiratory and Critical Care Medicine, Affiliated Hospital of Guilin Medical University. Simultaneously, 63 healthy controls were recruited from the Health Checkup Center of the Affiliated Hospital of Guilin Medical University. Healthy controls underwent pulmonary function tests. COPD patients fulfilled the diagnostic criteria for the Global Chronic Obstructive Pulmonary Disease Initiative (GOLD)^31^. To be included in the study, patients must show a ratio of forced expiratory volume in one second to forced vital capacity (FEV1/FVC) of less than 70% after inhalation of the β2-agonist (200 mg salbutamol). The definition of stable COPD is that the patient’s cough, expectoration, and shortness of breath are in stable condition or just show mild symptoms, or the condition is basically restored to the state before acute exacerbation^31^. Stable COPD patients’ severity is defined according to the results of lung function tests according to the GOLD criteria^31^. GOLD 1 (mild): FEV1 ≥ 80% predicted; GOLD 2 (moderate): FEV1 < 80% and ≥ 50% predicted; GOLD 3 (severe): FEV1 < 50% and ≥ 30% predicted; and GOLD 4 (very severe): FEV1 < 30% predicted. Exclusion criteria included: (1) treated with immunosuppressive drugs in the past month; (2) Acute respiratory infections within 4 weeks, such as pneumonia, acute and chronic bronchitis, and tuberculosis; (3) Combined with other respiratory diseases, such as asthma, bronchiectasis, and lung cancer; (4) Severe liver and renal insufficiency and cardiac insufficiency; (5) Pregnant or lactating women; (6) Severe mental disorders; (7) Patients that have other diseases that might impact the results of the current study.

### Lung function test

Pulmonary function tests were performed using a System 7 device (Minato Medical Science Co., Osaka, Japan) according to standards of the American Thoracic Society (ATS)/European Respiratory Society(ERS)^32^. The gender, age, height, and weight of participants were entered into the machine, and the expected value was automatically calculated. The Body Mass Index (BMI) was calculated as weight (kg) divided by height (cm) in square meters. The lung function parameters [forced vital capacity (FVC), percent of predicted FVC (FVC% predicted), forced expiratory volume in one second (FEV1), percent of predicted FEV1 (FEV1% predicted), the ratio of forced expiratory volume in one second to forced vital capacity (FEV1/FVC), forced expiratory flow 25% of FVC(FEF25), percent of predicted FEF25(FEF25% predicted), forced expiratory flow 50% of FVC(FEF50), percent of predicted FEF50(FEF50% predicted), maximal mid-expiratory flow (MMEF), and percent of predicted MMEF (MMEF % predicted)] were included for our analysis.

### Blood sample collection and analysis

Fasting blood samples were drawn, centrifuged, and serum was placed in plain polystyrene tubes on the same day. Serum samples were sent to the laboratory for storage at − 80 °C. blood neutrophil ratio and neutrophil counts were performed using a Sysmex XN2800 (Sysmex Co., Kobe, Japan) automatic blood cell analyzer on each participant. Serum concentrations of 14-3-3β protein in patients with stable COPD and healthy controls were measured by ELISA kits according to the manufacturer’s instructions (CUSABIO Life Science Ltd., Wuhan, China).

### PSM analysis

PSM analysis was performed in this study with SPSS software (version 22.0) and conducted with the 1:1 nearest neighbor matching method. The covariates included sex and age.

### Statistical methods

Statistical analysis was performed using SPSS software (version 22.0). Quantitative variables were presented as mean (standard deviation (SD)) or as the median and 25th-75th percentiles (interquartile range (IQR)). Categorical variables were presented as frequencies and percentages. Distribution normality was established by the Shapiro–Wilk normality test. Differences of sex between different groups were tested by the chi-square test. To compare two independent groups, the student *t*-test for normally distributed data or Mann–Whitney *U*-test (if a significant difference was seen for non-normally distributed variables) was employed to determine whether statistical significance existed. To compare more than 2 groups, the one-way analysis of variance (ANOVA) for normally distributed data (such as age) or Kruskal–Wallis *H*-test (if a statistical difference was observed for non-normally distributed variables) was used to determine whether statistical significance existed across the groups. The relationship of 14-3-3β protein to clinical parameters was assessed using the Pearson correlation coefficient or Spearman’s rank correlation coefficient. Receiver-operating characteristic (ROC) curves analysis was performed to evaluate the diagnostic utility of serum 14-3-3β protein. Significance was achieved when *P* < 0.05 for all tests.

## Supplementary Information

Supplementary Information 1.

## Data Availability

The data used to support the findings of this study are available from the corresponding author upon request.
