# Supplementary material for: Clinical significance of serum levels of 14-3-3β protein in patients with stable chronic obstructive pulmonary disease
Source: Sci Rep. 2023 Mar 24;13:4861. doi: 10.1038/s41598-023-32096-4 (PMC10039013; doi:10.1038/s41598-023-32096-4)
Supplement: Supplementary file 1 — Supplementary Information 1. [file 41598_2023_32096_MOESM1_ESM.docx]

**Clinical significance of serum levels of 14-3-3β protein in patients with stable chronic obstructive pulmonary disease**

**Decai Wang^1#^, Lizong Rao^4#^, Huiren Lei^2^, Wencui Li^2^, Qiufang Yu^2^, Wei Li^2^, Jianghong Wei^3^, Shuyun Xu^1^**⮹**, and Biwen Mo^2^**⮹

^1^ Department of Respiratory and Critical Care Medicine, Key Laboratory of Pulmonary Diseases of Health Ministry, Key Site of National Clinical Research Center for Respiratory Disease, Tongji Hospital, Tongji Medical College, Huazhong University of Science and Technology, Wuhan, 430030, Hubei, China.

^2^ Department of Respiratory and Critical Care Medicine, Guangxi Zhuang Autonomous Region Education Department Key Laboratory of Respiratory Diseases, Guangxi Health Commission Key Laboratory of Glucose and Lipid Metabolism Disorders, Second Affiliated Hospital of Guilin Medical University, Guilin, 541004, Guangxi, China.

^3^ Department of Respiratory and Critical Care Medicine, Affiliated Hospital of Guilin Medical University, Guilin, 541004, Guangxi, China.

^4^ Key Laboratory of Respiratory Disease of Zhejiang Province, Department of Respiratory and Critical Care Medicine, Second Affiliated Hospital of Zhejiang University School of Medicine, Hangzhou, 310000, Zhejiang, China.

**Supplementary Table 1 Serum levels of 14-3-3β protein in the COPD subgroup compared with healthy controls**

| Group, n | 14-3-3β (ng/mL) |
| --- | --- |
| HCs (63) | 25.77(18.09-34.41) |
| GOLD 1 (10) | 28.62(21.48-31.34) |
| GOLD 2 (21) | 36.05(29.84-44.38) ^*^ |
| GOLD 3＆4 (42) | 47.98(39.27-74.57) ^*#&^ |

Results are represented as n, or median (IQR). Data were tested by Kruskal-Wallis test. ^*^*P*<0.01 versus HCs; ^#^*P*<0.001 versus GOLD 1; ^&^*P*<0.05 versus GOLD 2; HCs: healthy controls; GOLD: Global Chronic Obstructive Pulmonary Disease Initiative.
